# Inactivating the spindle checkpoint kinase Bub1 during embryonic development results in a global shutdown of proliferation

**DOI:** 10.1186/1756-0500-2-190

**Published:** 2009-09-23

**Authors:** Valerie Tilston, Stephen S Taylor, David Perera

**Affiliations:** 1 Faculty of Life Sciences, University of Manchester, Michael Smith Building, Oxford Road, Manchester M13 9PT, UK; 2 MRC Centre for Drug Safety Science, Department of Pharmacology & Therapeutics, The University of Liverpool, Sherrington Building, Ashton Street, Liverpool L69 3GE, UK

## Abstract

**Background:**

Bub1 is a component of the spindle assembly checkpoint, a surveillance mechanism that maintains chromosome stability during M-phase. Bub1 is essential during the early stages of embryogenesis, with homozygous *BUB1*-null mice dying shortly after day E3.5. Bub1 is also required later during embryogenesis; inactivation of *BUB1 *on day E10.5 appears to rapidly block all further development. However, the mechanism(s) responsible for this phenotype remain unclear.

**Findings:**

Here we show that inactivating *BUB1 *on day E10.5 stalls embryogenesis within 48 hours. This is accompanied by a global shutdown of proliferation, widespread apoptosis and haemorrhaging.

**Conclusion:**

Our results suggest that Bub1 is required throughout the developing embryo for cellular proliferation. Therefore, Bub1 has been shown to be essential in all scenarios analyzed thus far in mice: proliferation of cultured fibroblasts, spermatogenesis, oogenesis and both early and late embryonic development. This likely reflects the fact that Bub1 has dual functions during mitosis, being required for both SAC function and chromosome alignment.

## Background

The correct segregation of chromosomes during mitosis and meiosis is crucial to ensure the maintenance of the ploidy of an organism. Loss or gain of a single chromosome during meiosis can cause birth defects, although more often it results in embryonic lethality [1]. Chromosome segregation errors during mitosis lead to aneuploidy, a common feature in multiple cancers. Correct chromosome segregation is normally ensured by the spindle assembly checkpoint (SAC), a signalling pathway which delays anaphase onset until all chromosomes are stably attached to spindle microtubules via their kinetochores [2]. The SAC consists of *"sensor" *proteins such as Bub1, Mad1 and Mps1, which in turn regulate *"signal transducers" *including BubR1, Bub3, Mad2 and Cdc20. The downstream *"effector" *of the SAC is the Anaphase Promoting Complex/Cyclosome (APC/C), an E3 ubiquitin ligase which targets for degradation proteins essential for mitotic exit such as Securin and Cyclin B1 [3].

The SAC is not essential in budding yeast [4,5], possibly because these cells enter mitosis with kinetochores already attached to microtubules [6]. In *Drosophila*, Mad2 mutants are also viable and fertile, possibly because correct chromosome attachment is also achieved very rapidly [7]. However, mutations in *BUBR1 *cause embryonic lethality in *Drosophila *[8]. This dichotomy raises the possibility that BubR1 may perform additional functions beyond simply delaying anaphase onset. Indeed, inhibition of BubR1 in human cancer cells inhibits chromosome alignment, even when anaphase onset is blocked downstream of the SAC [9].

In contrast to the situation in budding yeast and *Drosophila*, all the SAC genes studied to date are essential in mice. Embryos homozygous null for *MAD1*, *MAD2*, *BUB1*, *BUBR1 *and *BUB3 *die between embryonic days 3.5 and 8.5 [10-14]. This lethality may reflect the fact that embryonic divisions in the mouse are relatively fast during gastrulation [15] and are thus particularly dependent on the SAC. Interestingly however, two cell lines were derived from *MAD2*^-/-^*/p53*^-/- ^embryos harvested at day E10.5 [16]. This suggests that following the early embryonic divisions the SAC *per se *is not essential in mouse embryonic fibroblasts, mirroring the situation in *Drosophila*. Whether other SAC genes are essential following the early embryonic divisions is unclear as all the SAC mutant mice generated thus far are either constitutive knockouts or hypomorphs [10-13,17-19].

The one exception to this is Bub1. Using the Cre-LoxP system, we recently generated mice harbouring a conditional *BUB1 *gene [14]. We then crossed this strain with mice harbouring a transgene encoding a tamoxifen-responsive Cre recombinase [20]; this system allowed us to inactivate *BUB1 *in cultured mouse embryonic fibroblasts (MEFs), adult mice and developing embryos [14]. Bub1-deficient MEFs failed to align their chromosomes or sustain SAC function. Instead, they completed a single, highly aberrant mitosis and did not then divide again, probably because the aberrant mitosis triggered cell cycle arrest and/or apoptosis. Administration of tamoxifen into adult mice efficiently inactivated *BUB1 *in testes, causing sterility. Consistent with a potent anti-proliferative effect, mitotic cells and mature spermatids were virtually absent in Bub1-deficient seminiferous tubules. In female mice, using a ZP3-driven Cre to inactivate *BUB1 *during oogenesis results in premature extrusion of the first polar body and massive chromosome missegregation during meiosis I [21]. To determine the requirement for Bub1 during the later stages of embryonic development, we injected tamoxifen into pregnant females carrying conditional *BUB1 *embryos, 10.5 days post coitum (dpc). The litters were then harvested on day 18.5 pc. Strikingly, Bub1-deficient embryos were highly abnormal, resembling embryos normally found at day E10.5-11.5, indicating a very rapid block to embryogenesis.

One possibility is that this defect is due to an abnormality in a specific tissue. For example, when a tamoxifen-responsive Cre was used to inactivate the von-Hippel-Lindau (VHL) tumor suppressor during late embryonic development, the VHL-deficient embryos died six days after administration of tamoxifen due to severe liver damage [22]. Alternatively, because Bub1 is essential in MEFs, the block on embryogenesis could be due to a global proliferative defect. Here, we describe Bub1-deficient embryos in more detail and show that the catastrophic developmental defect is most likely due to a global shutdown of proliferation defect and widespread apoptosis.

## Results and Discussion

**Figure 1:**
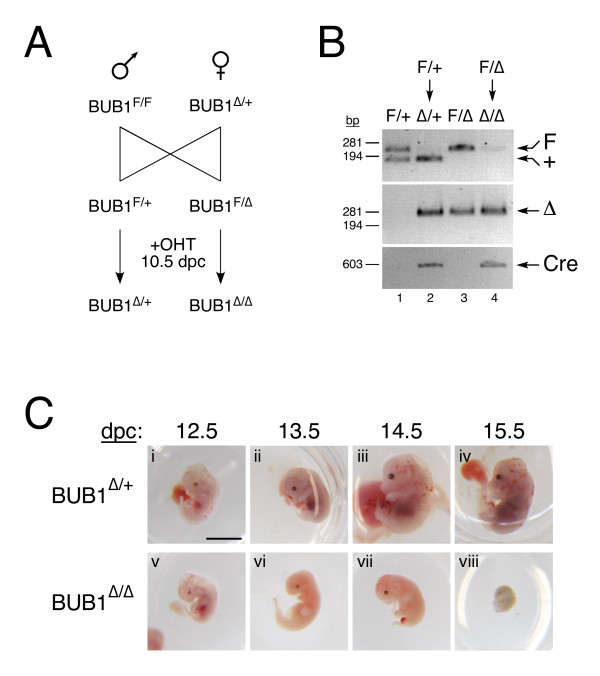
**Mid-gestation inactivation of *BUB1 *arrests embryonic development**. **(A) **Schematic of mating regimen. A *BUB1*^*F*/*F *^*ER*^*T*^-*Cre *male was crossed with a *BUB1*^Δ/+ ^*ER*^*T*^-*Cre *female. The pregnant female was injected with tamoxifen at 10.5 dpc, then the embryos were harvested and genotyped at various times after injection. **(B) **PCR genotyping of 12.5-dpc embryos to determine the *BUB1 *genotype (top and middle panels) and to detect the presence of *Cre *(lower panel). See main text for details. **(C) **Images of *BUB1*^Δ/+ ^embryos (panels *i *to *iv*) and *BUB1*^Δ/Δ ^embryos (panels *v *to *viii*) after harvesting on the indicated day post coitum, after removal of the placenta and yolk sac. Scale bar, 5 mm.

Using homologous recombination in mouse ES cells, we previously flanked exons 7 and 8 of the *BUB1 *gene - which encode the Bub3-binding domain [23] - with *LoxP *sites to create the floxed allele *BUB1*^*F *^(flanked by LoxP) [14]. Mice harbouring this allele were then crossed with a Cre-deleter strain to create the null allele, *BUB1*^Δ ^[14]. To analyse the effects of inactivating *BUB1 *in late stages of embryonic development, we crossed *BUB1*^*F*/*F *^males with *BUB1*^Δ/+ ^females, both harbouring the *ER*^*T*^-*Cre *transgene. This mating generated embryos that were either *BUB1*^*F*/+ ^or *BUB1*^*F*/Δ ^(Figure 1A). Pregnant females were then injected with tamoxifen at 10.5 dpc, thereby converting the embryo's floxed alleles to null alleles, i.e. generating *BUB1*^Δ/+^and *BUB1*^Δ/Δ ^embryos (Figure 1A). Importantly, tamoxifen should have no effect on the pregnant females because they lack floxed alleles. Moreover, after administration of tamoxifen, the *BUB1*^Δ/+ ^embryos still retained one wild-type *BUB1 *allele (Figure 1A). Because Bub1 heterozygotes are normal [14], these embryos served as an internal negative control. Two to five days following tamoxifen injection the uterus was dissected and the embryos, placenta and yolk sac processed for analysis.

PCR genotyping of 12.5-dpc *BUB1*^*F*/+ ^embryos lacking the *ER*^*T*^-*Cre *transgene showed the presence of both floxed and wildtype alleles (Figure 1B, lane 1). By contrast, the floxed allele was barely detectable in *BUB1*^*F*/+ ^embryos harbouring *ER*^*T*^-*Cre *after tamoxifen injection, but they were positive for the null allele, indicating efficient recombination at the *BUB1*^*F *^allele (Figure 1B, lane 2). Similarly, the floxed allele was barely detectable in *BUB1*^*F*/Δ ^embryos harbouring *ER*^*T*^-*Cre *demonstrating efficient deletion of the floxed allele (Figure 1B, compare lanes 3 and 4).

Examination of the placenta and yolk sac at 12.5 dpc revealed no dramatic morphological changes between *BUB1*^Δ/+ ^and *BUB1*^Δ/Δ ^embryos (data not shown). However, once the placenta and yolk sac were removed, closer inspection of the embryos showed that 12.5-dpc *BUB1*^Δ/Δ ^embryos were slightly smaller than their *BUB1*^Δ/+ ^littermates (Figure 1C, compare panels *i *and *v*). This phenotype became much more apparent from 13.5 dpc onwards, with the *BUB1*^Δ/Δ ^embryos appearing much paler, smaller and underdeveloped than their *BUB1*^Δ/+ ^littermates (Figure 1C). By 15.5 dpc, the *BUB1*^Δ/Δ ^embryos had completely degenerated and their size was comparable to embryos at E10.5-11.5 (Figure 1C, compare panels *iv *and *viii*).

**Figure 2:**
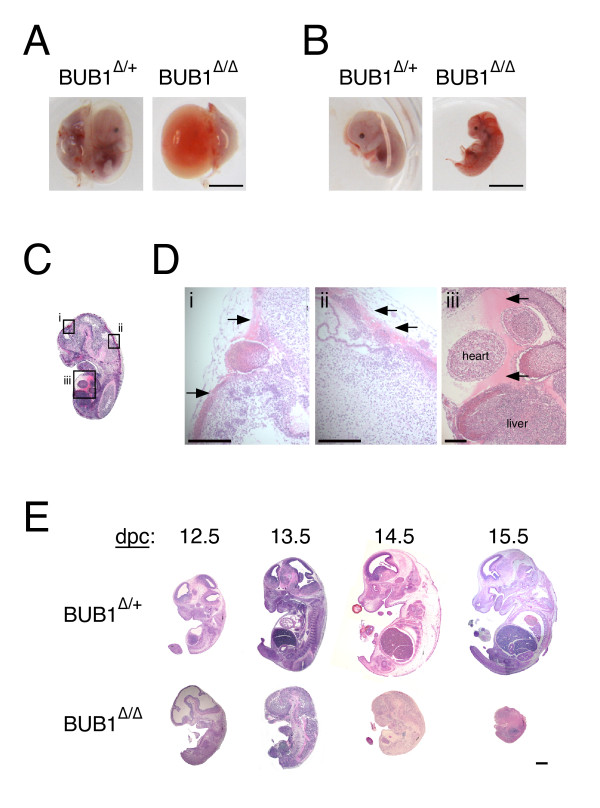
**Histological analysis of BUB1Δ/+ and BUB1Δ/Δ embryos**. **(A) **Images of *BUB1*^Δ/+ ^and *BUB1*^Δ/Δ ^embryos harvested 3 days after tamoxifen injection. Scale bar, 5 mm. **(B) **Images of same embryos as in A, after removal of the placenta and yolk sac. Scale bar, 5 mm. **(C) **Sagittal section of the same *BUB1*^Δ/Δ ^embryo as in A and B, stained with haematoxylin-eosin, showing signs of extensive haemorrhaging. **(D) **Enlargements of regions marked in C, showing haemorrhaging (arrows) in the brain area (panel *i*), the spinal cord (panel *ii*) and the body cavity (panel *iii*). Scale bars, 0.5 mm.**(E) **Embryos were harvested at 2-5 days after tamoxifen injection, fixed, then sections prepared and stained with haematoxylin-eosin. Scale bar, 1 mm.

Frequently, haemorrhaging was observed within the yolk sac of *BUB1*^Δ/Δ ^embryos at 13.5 dpc (Figures 2A). Similarly, the embryos themselves showed signs of haemorrhaging (Figure 2B). Histological analysis of a 13.5-dpc *BUB1*^Δ/Δ ^embryo by haematoxylin-eosin staining revealed extensive haemorrhaging within the embryo, around the periphery, and especially within the brain and body cavity (Figures 2C and 2D; see Additional File 1). Taken together, these results indicate that *BUB1*^Δ/Δ ^embryos undergo developmental arrest between 2 and 3 days after tamoxifen injection, and probably die due to global haemorrhaging.

Histological analysis of the embryos by haematoxylin-eosin staining revealed stunted development and severe tissue deterioration in *BUB1*^Δ/Δ^embryos (Figure 2E). At 12.5 dpc, the tissue structure of *BUB1*^Δ/Δ ^embryos appeared less defined than their *BUB1*^Δ/+ ^littermates, with some underdeveloped areas, especially within the brain. By 13.5 dpc, this defective tissue structure was more readily apparent, and the development of *BUB1*^Δ/Δ^embryos was clearly retarded. As *BUB1*^Δ/+ ^embryos developed further, their *BUB1*^Δ/Δ ^littermates appeared much smaller and massively degenerated (Figure 2E).

**Figure 3:**
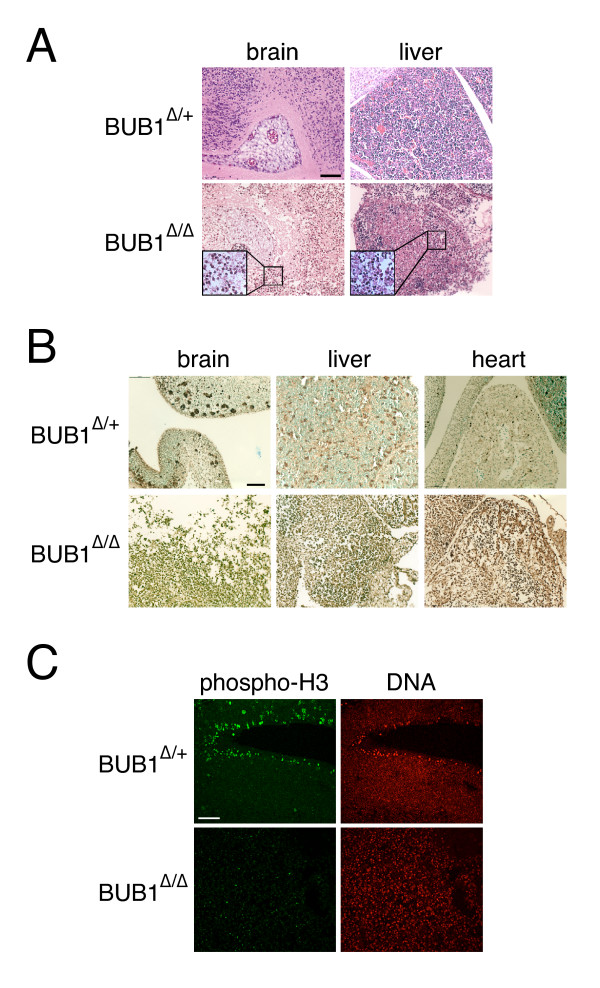
**Decreased mitotic index and apoptosis in Bub1-null embryos**. **(A) **Haematoxylin-eosin-stained sections of brain and liver from embryos harvested 3 days after tamoxifen injection. Enlargements show numerous pyknotic nuclei in tissues from *BUB1*^Δ/Δ ^embryos. Scale bar, 0.2 mm. **(B) **Massive apoptosis caused by *BUB1 *inactivation. *BUB1*^Δ/+ ^and *BUB1*^Δ/Δ ^embryos were harvested 3 days after tamoxifen injection, fixed, then sections prepared and stained to detect DNA fragmentation. Scale bar, 0.2 mm. **(C) **Reduced number of mitotic cells in Bub1-deficient embryos. Embryo sections prepared as in (B) were stained to detect phospho-histone-H3 (green) and DNA (red). Scale bar, 0.5 mm.

One possible explanation for the fast and dramatic developmental arrest observed in *BUB1*^Δ/Δ ^embryos is a massive apoptotic response being triggered shortly after *BUB1 *inactivation. Indeed, sections of brain and liver stained with haematoxylin-eosin revealed the presence of multiple pyknotic nuclei, a hallmark of apoptotic death, in *BUB1*^Δ/Δ ^embryos (Figure 3A). We therefore decided to look at the extent of apoptosis shortly after tamoxifen-mediated *BUB1 *inactivation. For that purpose, sagittal sections of 13.5-dpc embryos were stained to detect DNA fragmentation, a well-described characteristic of apoptosis (see Additional File 1). Sections of *BUB1*^Δ/+ ^embryos display normal tissue structure with green-stained viable cells and few clusters of apoptotic dark brown-stained cells (Figure 3B). By contrast, *BUB1*^Δ/Δ ^embryos are predominantly stained dark brown, suggesting extensive apoptosis. Closer inspection of the brain, liver and heart revealed a large increase in the number of apoptotic cells within *BUB1*^Δ/Δ ^embryos compared to their control littermates (Figure 3B). These results confirm that the loss of tissue structure observed in Bub1-depleted embryos is highly likely due to increased levels of apoptotic cell death.

When *BUB1 *is inactivated in cultured MEFs and adult mouse testes, aberrant mitoses occur which in turn stops further cell proliferation [14], probably due to the accumulation of extensive genomic damage which in turn activates p53-dependent pathways. Consequently, after cells have completed one cell cycle in the absence of Bub1, they rarely enter an additional mitosis, in turn leading to a low mitotic index [14]. To determine if a similar phenomenon was occurring in the Bub1-deficient embryos, we stained embryo sections with an antibody that recognises histone H3 when phosphorylated on serine 10, a mitosis-specific modification (see Additional File 1). Discrete clusters of phospho-histone H3 positive cells were observed in the brains of 13.5-dpc *BUB1*^Δ/+ ^embryos (Figure 3C) as well as the snout, liver, hind limbs and spine (data not shown). Importantly, few if any phospho-histone H3 positive cells were observed in the *BUB1*^Δ/Δ ^embryos (Figure 3C). These data therefore indicate that three days after injecting tamoxifen there are very few mitotically active cells in the Bub1-deficient embryos, indicating that inactivation of *BUB1 *inhibits further proliferation.

Taken together, our observations indicate that Bub1 is not only essential during early embryonic development, but also during the later stages of embryogenesis. Indeed, inactivation of Bub1 mid-way through gestation blocks late embryonic development due to a global shutdown of proliferation rather than a specific defect in a particular tissue. The observed catastrophic development of Bub1-deficient embryos is more severe than one might expect due to a SAC defect. In principle, the SAC monitors the chromosome alignment process and delays anaphase until all the chromosomes are correctly attached to the spindle [2]. In the absence of SAC function, mitosis normally lasts long enough for most cells to accurately segregate their chromosomes [24]. This is demonstrated most clearly by the fact that SAC genes are not essential in budding yeast [4,5] and that Mad2-deficient flies are viable and fertile [7]. Although Bub1 is required for SAC function in mice [14], the severity of the defect during mouse development is unlikely to reflect solely a SAC defect. Indeed, down-regulation of Bub1 by RNA interference in human tissue culture cells and *BUB1 *inactivation in MEFs cause extensive chromosome misalignment [14,19,25,26]. This may mean that Bub1 plays two separate roles, one in the SAC and another in regulation of kinetochore-microtubule interactions, as opposed to Mad2, which is a core component of the SAC pathway but has no function in chromosome alignment. Alternatively, perhaps removing a key SAC component from the kinetochore has knock-on consequences for kinetochore function such that correct microtubule interactions are now no longer possible. Distinguishing between these two scenarios will require separation-of-function mutants. Nevertheless, combining a SAC defect with a chromosome alignment defect clearly has dramatic consequences, not only in flies as shown by the lethality caused by BubR1 mutation, but also during mouse development, as shown here by inactivating *BUB1*.

## Competing interests

The authors declare that they have no competing interests.

## Authors' contributions

The project was conceived by S.S.T., the experiments performed by V.T. and D.P., the manuscript was written by D.P. All authors read and approved the final manuscript.

## Supplementary Material

Additional file 1 **Materials and Methods**. Details about the methodology used in this paper: Matings and genotyping; Histological Analysis; Immunofluorescence; Apoptosis Assay. Click here for file
