# Supplementary material for: Inactivating the spindle checkpoint kinase Bub1 during embryonic development results in a global shutdown of proliferation
Source: BMC Res Notes. 2009 Sep 23;2:190. doi: 10.1186/1756-0500-2-190 (PMC2754486; doi:10.1186/1756-0500-2-190)
Supplement: Additional file 1 — Materials and Methods. Details about the methodology used in this paper: Matings and genotyping; Histological Analysis; Immunofluorescence; Apoptosis Assay. [file 1756-0500-2-190-S1.DOC]

### MATERIALS AND METHODS

**Matings and genotyping**

Mice harbouring conditional and null *BUB1* alleles and the *ER^T^-Cre* transgene were as described [1]. Timed matings were set up and at 10.5 dpc pregnant females were injected intraperitoneally with tamoxifen (Sigma; 5 mg per 40 g body weight) dissolved in corn oil (Sigma). Females were sacrificed and embryos collected 2-5 days after tamoxifen injection. Embryos and their placentas were photographed and part of the embryo tail removed for genotyping before the remaining embryo was fixed for histological analysis. Preparation of genomic DNA and PCR genotyping was as described [1]. All mice were hosted in a pathogen-free facility at the University of Manchester.

**Histological analysis**

Embryos were fixed overnight in Bouin’s solution and processed for paraffin sectioning. 5µm-thick paraffin sections were deparaffinized, rehydrated and stained with haematoxylin/eosin. Sections were analysed and images collected on an Axioskop upright microscope using an Axiocam colour CCD camera and Axiovision software.

**Immunofluorescence**

5µm-thick paraffin sections were deparaffinized, rehydrated and blocked in PBS plus 5% BSA before staining with rabbit anti-phospho-histone H3 antibody (1:200; Millipore) overnight at 4°C. Following washes in PBS, sections were stained with Alexa488-conjugated secondary antibody (1:500; Molecular Probes), stained with Hoechst 33358 (Sigma), and then mounted. Images were taken on a Leica TCS SP5 AOBS inverted confocal microscope.

**Apoptosis assay**

13.5-dpc embryos were fixed overnight in 10% Neutral Buffered Formalin and processed for paraffin sectioning. 5µm-thick paraffin sections were collected on Poly-L-Lysine-coated slides and processed as per manufacturer’s instructions using TdT-FragEL DNA Fragmentation Detection Kit (Calbiochem). Sections were analysed and images collected on an Axioskop upright microscope using an Axiocam colour CCD camera and Axiovision software.

**REFERENCES (Methods Section)**

1. Perera D, Tilston V, Hopwood JA, Barchi M, Boot-Handford RP, Taylor SS: **Bub1 maintains centromeric cohesion by activation of the spindle checkpoint**. *Dev Cell* 2007, **13**(4):566-579.
